# A Muscle-Preserving Short Transverse Incision for Unicompartmental Knee Arthroplasty: A Technical Note

**DOI:** 10.7759/cureus.43662

**Published:** 2023-08-17

**Authors:** Sho Tanaka, Takafumi Hiranaka, Yasuhiro Fukai, Takahiro Okajima, Tatsuhiko Kanno

**Affiliations:** 1 Orthopaedic Surgery and Joint Surgery Centre, Takatsuki General Hospital, Takatsuki, JPN

**Keywords:** transverse, incision, unicompartmental, arthroplasty, technique, surgery, knee

## Abstract

We describe the use of a short transverse incision technique with muscle retention for unicompartmental knee arthroplasty (UKA). The incision is made transversely just above the joint line, followed by a detachment of subcutaneous soft tissue from the underlying capsule and fascia to create a mobile window. The fascia is incised along the medial border of the vastus medialis and the capsule of the suprapatellar pouch is incised laterally, preserving vastus medialis muscle. All procedures are performed within the mobile window while controlling the knee flexion angle. Following implantation, the capsule and fascia are anatomically repaired. This approach was used in 30 consecutive patients who underwent Oxford UKA, including one bi-unicompartmental knee arthroplasty without complications. Importantly, no patients had any disturbances of the infrapatellar branch of the saphenous nerve disturbances such as numbness, hyperesthesia, hypoesthesia, or neuroma pain. The transverse approach is thought to be a safe and feasible method for UKA.

## Introduction

Unicompartmental knee arthroplasty (UKA) is a treatment option for monocondylar osteoarthritis. However, it is associated with several complications that may require revision surgery, including progression of osteoarthritis in the lateral compartment, tibial fracture, and dislocation of the mobile bearing [1]. Although infrapatellar branch of the saphenous nerve (IPBSN) injury has typically been considered trivial, it can cause numbness, discomfort, and painful neuroma, and thus can decrease patient satisfaction after total knee arthroplasty (TKA) [2]. As the location of the IPBSN has been reported as 17 mm to 46 mm from the tibial tuberosity [3], or 28 mm distal from the inferior pole of the patella [4], it can be encountered in UKA as long as a longitudinal incision is made. A transverse incision can avoid IPBSN damage because it runs parallel to the nerve, but it requires approximately 15 cm incision for TKA, and the nerve disturbance cannot be completely prevented [5]. Meanwhile, in UKA, such a transverse incision can be facilitated because UKA requires a comparatively short incision. Moreover, muscle retention is an important factor for quick recovery. Here, we demonstrate a technique of short transverse incision with muscle retention for Oxford UKA.

## Technical report

A leg-hanging position or supine position is used. A 4 cm transverse incision is made approximately 5 mm above the joint line. The medial incision line goes slightly upward, following the course of the IPBSN. The soft tissue is bluntly dissected up to the ligamentous capsule, the subcutaneous soft tissues are then detached from the underlying capsule, patellar and patellar-tendon retinaculum, tibial periosteum, and quadriceps fascia so that the medial aspect of the knee is exposed, and the skin opening is mobile. As described in a previous report, the joint is opened in a manner that retains the vastus medialis muscle with several minor modifications [6].

**Figure 1:**
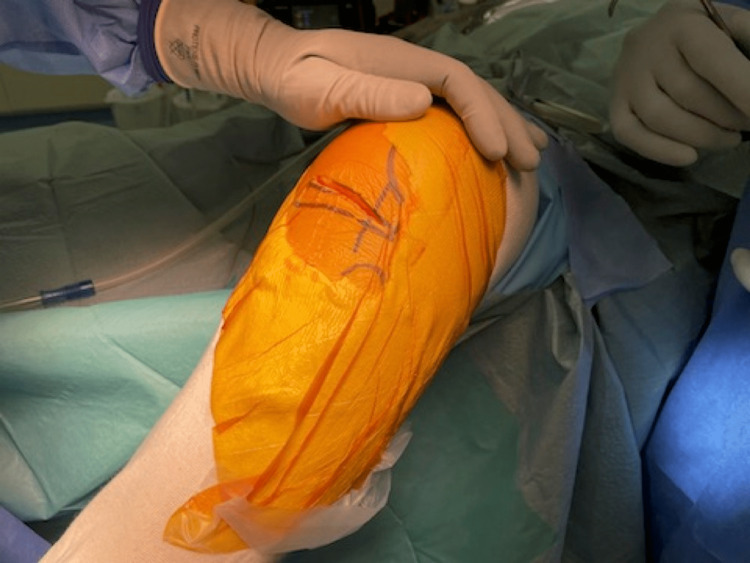
The capsulotomy is made from 1 cm below the joint line to the insertion of the vastus medialis muscle to the patella, along the medial border of the patella and patella tendon.

**Figure 2:**
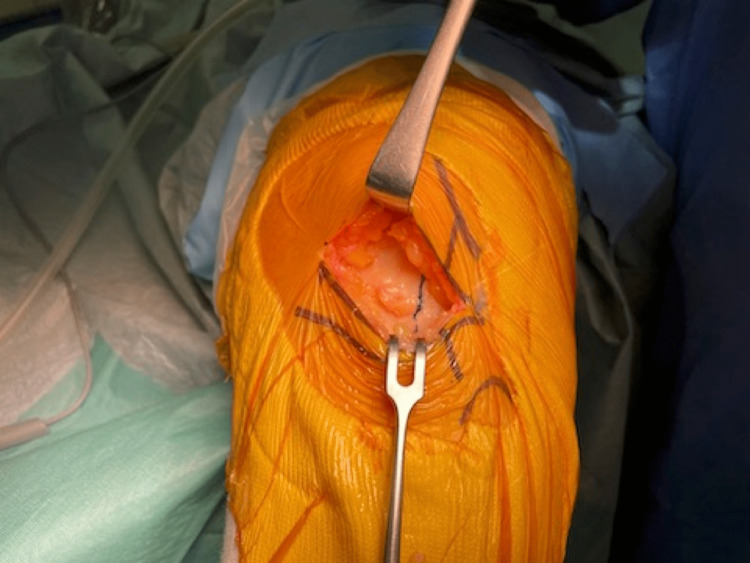
The capsulotomy directs medially, followed by fasciotomy along the medial border of the vastus medialis.

The capsulotomy is made from 1 cm below the joint line to the insertion of the vastus medialis muscle to the patella, along the medial border of the patella and patella tendon (Figure 1). Then, the capsulotomy directs medially, followed by fasciotomy along the medial border of the vastus medialis (Figure 2).

Good knowledge of the anatomy around this area is necessary. The deepest layer comprises the synovium membrane of the knee joint. It extends as the thick capsule of the suprapatellar pouch proximally and as the very thin capsule covering Hoffa's fat pad. The medial patellofemoral ligament is often found as a thick band at the superomedial corner of the patella level. The superior layer comprehensively comprises the medial patellar retinaculum and fascia over the vastus medialis muscle. The fascia thickens medially and distally, connecting to the medial intramuscular septum and suprapatellar pouch, respectively, and forming the intermediate layer.

**Figure 3:**
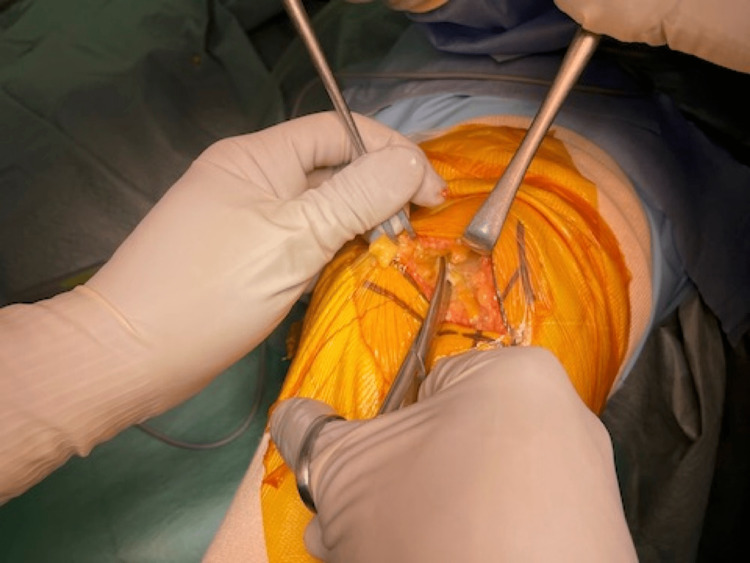
The muscle belly is then detached from the surrounding fascia to be retracted in a lateral direction.

**Figure 4:**
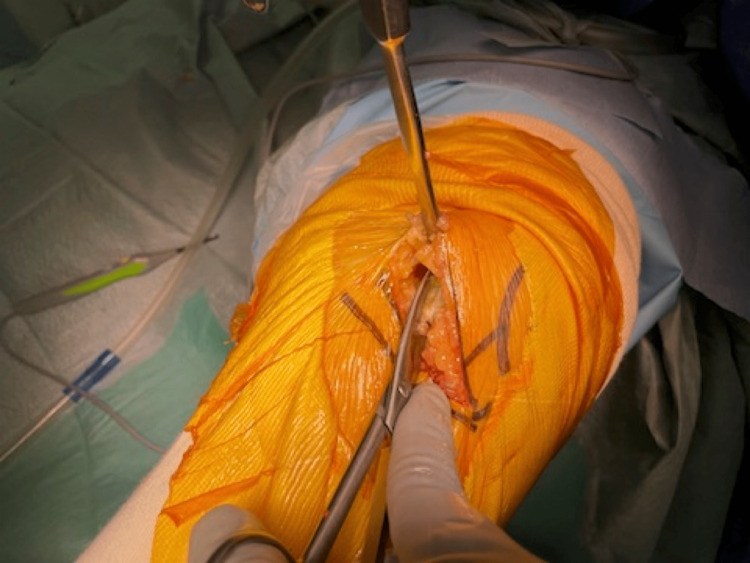
The intermediate layer that separates the muscle compartment and the joint cavity is then exposed, and the septum is exposed so that the patellar can be retracted in a lateral direction.

The fasciotomy following the capsulotomy is made just lateral to the medial intramuscular septum and 2 to 3 cm proximally. The muscle belly is then detached from the surrounding fascia to be retracted in a lateral direction (Figure 3). The intermediate layer that separates the muscle compartment and the joint cavity is then exposed, and the septum is exposed so that the patellar can be retracted in a lateral direction (Figure 4).

The inelastic tissues, such as the capsule and fascia, are incised, whereas the muscle is stretchable, so the patella can be retracted even if it is retained. The capsular cut should be made as lateral as possible for easier retraction of the patella.

**Figure 5:**
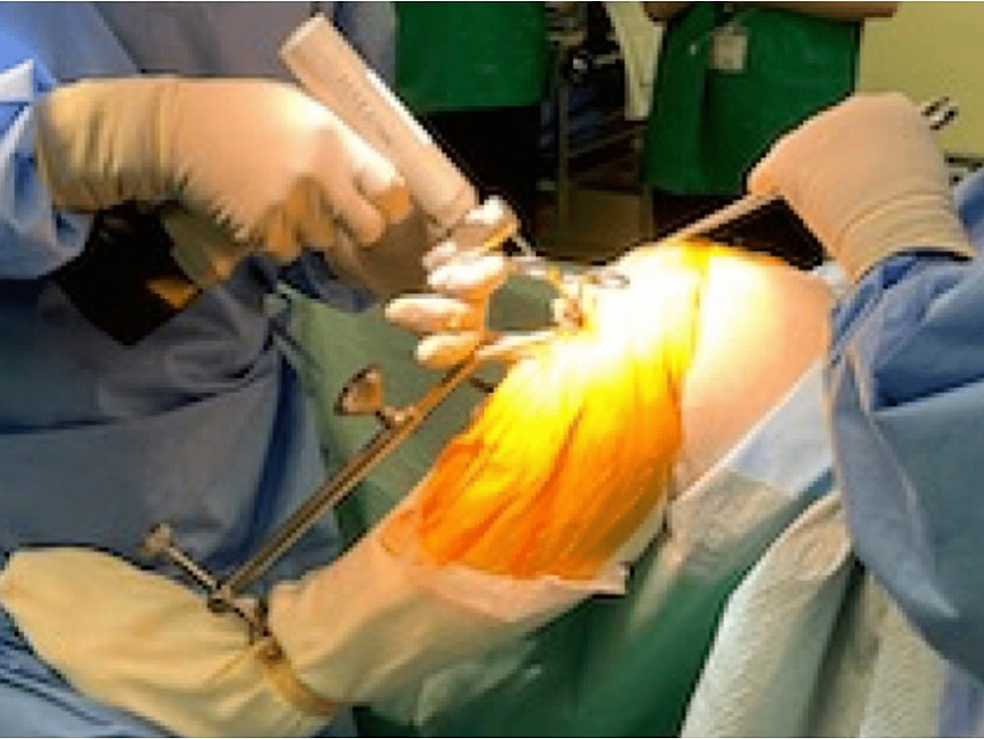
A deep knee flexion facilitates access to the femoral side, and mid-flexion eases the maneuver of the tibial side.

The later steps of the procedure can be made following the manufacturer-provided operation manual [7]. By detaching the subcutaneous soft tissues from the underlying fascia and capsule, the mobile window helps get good visualization. Knee flexion angle also affects visualization. A deep knee flexion facilitates access to the femoral side, and mid-flexion eases the maneuver of the tibial side (Figure 5).

**Figure 6:**
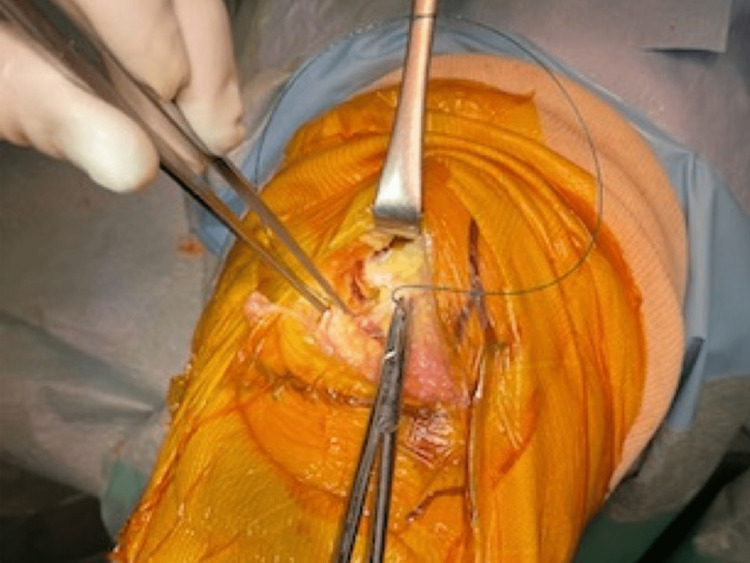
The capsule of the suprapatellar pouch is sutured, followed by the closure of the knee joint capsule.

**Figure 7:**
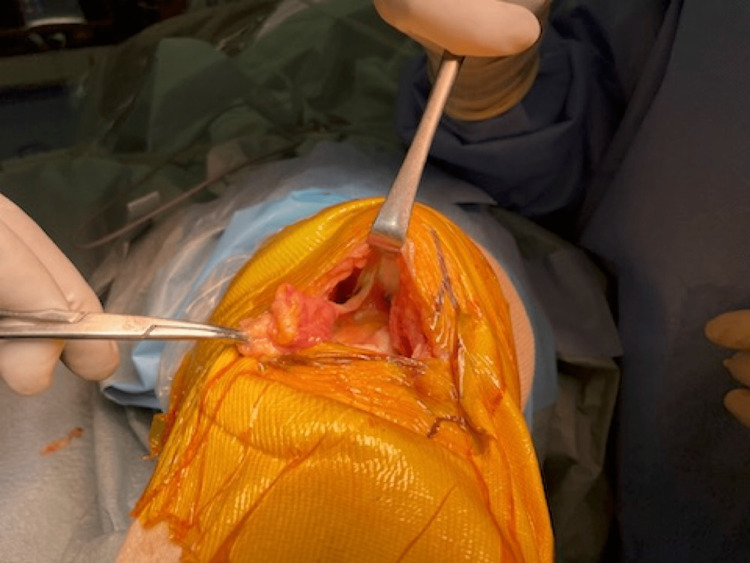
The capsule of the suprapatellar pouch is sutured.

**Figure 8:**
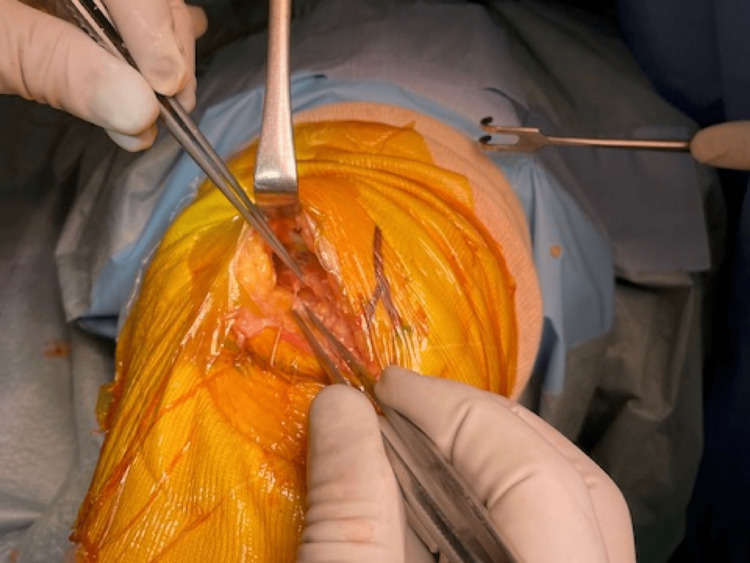
At the lower end of the muscle, the fascia thickens and provides medial force for the patella.

**Figure 9:**
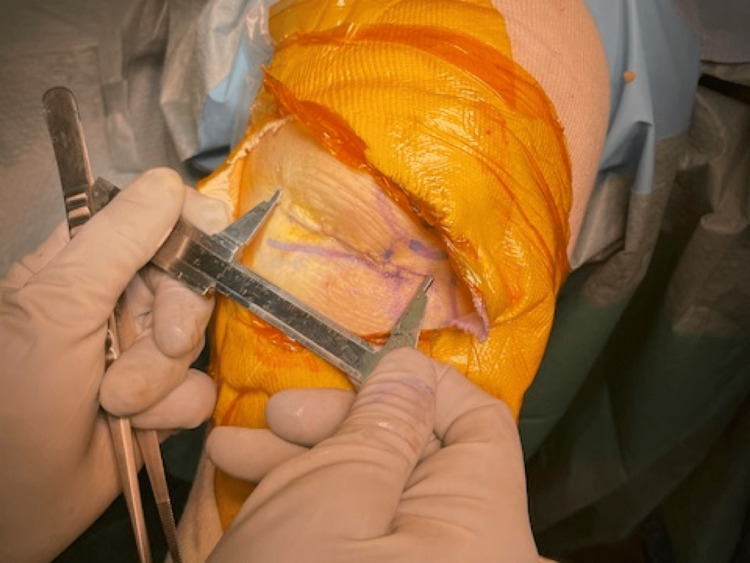
After suturing the fascia, the skin is closed.

After the implantation, the capsule of the suprapatellar pouch is sutured (Figure 6), followed by the closure of the knee joint capsule (Figure 7), and then the repair of the fascia over the vastus medialis. At the lower end of the muscle, the fascia thickens and provides medial force for the patella (Figure 8), so it must be repaired tightly. After suturing the fascia, the skin is closed (Figure 9).

The average operation time was 46 min for a single-side operation and 88 min for a simultaneous bilateral operation (UKA only). There were no wound-related complications. Notably, no patients had neurogenic pain numbness, hyperesthesia, or hypoesthesia on the anteromedial aspect of the leg.

## Discussion

A transverse incision for UKA is reported here for the first time. No cases required conversion of the incision, regardless of the body size. Our case series included all Japanese patients but included some male patients whose body size was equivalent to the average Western population, and the procedure could be done without difficulty. The key point of this approach is the detachment of the soft tissue from the underlying fascia and capsule. It seems that this approach may be used for any patient undergoing UKA.

In our series, no sensory disturbance or neurogenic pain occurred owing to an IPBSN injury. This might be due to the incision being made parallel to the IPBSN. Once the incision is made along the IPBSN and the soft tissue that might include the IPBSN is detached from the capsule, there is virtually no chance of encountering the nerve in the operative field. On the other hand, the nerve may be encountered if a longitudinal incision is used [8]. The transverse approach did not cause wound trouble within acceptable operation times. Another benefit of this approach is that the cut is parallel to the skin crease (skin-crease incision). The benefits of skin-crease incisions compared with longitudinal incisions have been shown in several reports; perhaps the most important benefit of skin-crease incisions is the cosmetic result [9]. Moreover, the longitudinal incision is likely beneficial in terms of pain and limitation of flexion due to the tightness of the scar. Although a comparative study is needed, the skin-crease incision seems to be of benefit to patients that undergo UKA. Muscle retention and anatomical closure of the capsule and fascia were possible despite the short incision length. Numerous studies have shown the benefits of muscle-retention approaches, such as the subvastus and quadriceps-sparing approaches [10], although the approaches have been longitudinal. Moreover, very few reports have described soft tissue repair [6]. In addition to muscle retention, anatomical capsule closure can reduce operative invasiveness, such as leakage of the intra-articular bleeding to the muscle compartment, which can cause muscle inflammation followed by pain, swelling, and loss of flexion.

There are potential limitations to this approach. First, we applied it only to UKA, not to TKA. There is one report of transverse incision for TKA [5], so adapting this approach to TKA might be possible, but a sensory disturbance was found in approximately 30% of their patients [5]. As our series did not show such a complication, applying the transverse approach to TKA must be deliberate even though it might be feasible. Second, it is also difficult to extend the incision if revision surgery is required. Fortunately, we have thus far had no cases requiring revision, but the incision for the revision surgery should be considered in advance. As the incision is very small and parallel to the skin crease, the cosmetic results are favorable, and a conventional longitudinal incision can be used for revision surgery. Although this technique is beneficial for both reducing invasiveness and IPBSN injury, it is technically demanding. Therefore, this approach should be done after the traditional procedure is well-mastered. Lastly, this report is simply a technical note aimed at showing the feasibility of the approach, a comparable study with long-term follow-up is required to prove the advantages of this approach.

## Conclusions

In conclusion, the transverse approach is thought to be feasible and safe in patients undergoing UKA. Notably, there were no patients with a sensory disturbance or neuroma. Moreover, muscle retention and anatomical soft tissue closure are also possible through this approach. Further study is required to prove the benefit of this approach over the conventional longitudinal approach.
